# Galectin-3 Decreases 4-1BBL Bioactivity by Crosslinking Soluble and Membrane Expressed 4-1BB

**DOI:** 10.3389/fimmu.2022.915890

**Published:** 2022-06-24

**Authors:** Morten Aagaard Nielsen, Kristian Juul-Madsen, John Stegmayr, Chao Gao, Akul Y. Mehta, Stinne Ravn Greisen, Tue Wenzel Kragstrup, Malene Hvid, Thomas Vorup-Jensen, Richard D. Cummings, Hakon Leffler, Bent Winding Deleuran

**Affiliations:** ^1^ Department of Biomedicine, Aarhus University, Aarhus, Denmark; ^2^ Department of Rheumatology, Aarhus University Hospital, Aarhus, Denmark; ^3^ Department of Experimental Medical Sciences, Faculty of Medicine, Lund University, Lund, Sweden; ^4^ Wallenberg Centre for Molecular Medicine, Faculty of Medicine, Lund University, Lund, Sweden; ^5^ Stem Cell Center, Faculty of Medicine, Lund University, Lund, Sweden; ^6^ Division for Microbiology, Immunology and Glycobiology (MIG), Department of Laboratory Medicine, Lund University, Lund, Sweden; ^7^ Department of Surgery, Beth Israel Deaconess Medical Center, Harvard Medical School, National Center for Functional Glycomics, Boston, MA, United States; ^8^ Department of Clinical Medicine, Aarhus University, Aarhus, Denmark

**Keywords:** checkpoint receptor, 4-1BB, Galectin-3, inflammation, rheumatoid arthritis

## Abstract

4-1BB is a T cell costimulatory receptor and a member of the tumor necrosis factor receptor superfamily. Here, we show that Galectin-3 (Gal-3) decreases the cellular response to its ligand (4-1BBL). Gal-3 binds to both soluble 4-1BB (s4-1BB) and membrane-bound 4-1BB (mem4-1BB), without blocking co-binding of 4-1BBL. In plasma, we detected complexes composed of 4-1BB and Gal-3 larger than 100 nm in size; these complexes were reduced in synovial fluid from rheumatoid arthritis. Both activated 4-1BB^+^ T cells and 4-1BB-transfected HEK293 cells depleted these complexes from plasma, followed by increased expression of 4-1BB and Gal-3 on the cell surface. The increase was accompanied by a 4-fold decrease in TNFα production by the 4-1BB^high^Gal-3^+^ T cells, after exposure to 4-1BB/Gal-3 complexes. In RA patients, complexes containing 4-1BB/Gal-3 were dramatically reduced in both plasma and SF compared with healthy plasma. These results support that Gal-3 binds to 4-1BB without blocking the co-binding of 4-1BBL. Instead, Gal-3 leads to formation of large soluble 4-1BB/Gal-3 complexes that attach to mem4-1BB on the cell surfaces, resulting in suppression of 4-1BBL’s bioactivity.

## Introduction

Orchestration of immune checkpoints is central for the outcome of immune activation, especially in patients with chronic inflammation and cancer. 4-1BB (CD137/TNFRSF9) plays a major role in regulating the outcome of the adaptive immune system (1–3). 4-1BB is a promising interventional target in severe autoinflammatory disease such as rheumatoid arthritis (RA). Despite the central role in shaping an adaptive immune response, effective treatment strategies utilizing agonistic 4-1BB antibodies has been challenging. As suggestive of that, complex local modulatory mechanisms are important for the function of 4-1BB (4–7).

4-1BB is a glycosylated co-stimulatory transmembrane receptor expressed on a range of immune cells (8), but predominantly expressed on T cells upon antigen encounter (9). 4-1BB signaling promotes clonal expansion, accumulation of antigen-specific effector CD4+ and CD8+ T cells, and formation of proinflammatory cytokines (10, 11). 4-1BB ligand (4-1BBL) is the cognate ligand for 4-1BB and is mainly expressed by activated antigen-presenting cells (APC) (12, 13). In humans, 4-1BB loss-of-function mutations led to impaired T cell activation, proliferation, and differentiation, highlighting the pivotal role of functional 4-1BB for immune homeostasis (14). Additionally, it has become evident that glycans and glycan-binding proteins are also of key importance in 4-1BB signaling (15, 16), however the wider implications for immune regulation are unclear.

Galectins are a family of widely expressed glycan-binding proteins, defined by their shared carbohydrate recognition domain (CRD) and their differential affinities for β-galactosides and related glycans (17). Galectins are expressed by several immune cells involved in inflammation including macrophages, lymphocytes and stromal cells, where they modulate the inflammatory response by interaction with multiple glycosylated receptors (18–21). Thus, galectins are capable of modulating inflammation both locally and systemically.

We, and other investigators, have shown that galectin-9 (Gal-9) is important for 4-1BB signaling leading to production of pro-inflammatory cytokines in RA. This occurs independent of Gal-9 interfering with the ability of ADAM-17 to cleave 4-1BB from the cell membrane (15, 16). Apart from Gal-9, several other galectins are also linked to RA pathology (22–25). Of the eleven known members of the human galectin family, galectin-3 (Gal-3) is structurally unique, since it is the only chimera-type galectin which contains an N-terminal peptide domain that can form oligomers upon interactions with ligands (26). However, the mechanistic involvement of Gal-3 in chronic inflammation is only partially understood (27).

Based on the above, we therefore hypotheses that Gal-3’s could influence 4-1BB function in RA. We report that Gal-3 is a novel carbohydrate-dependent binding partner of 4-1BB, and through this interaction induces opposite reactions compared to those observed by Gal-9 in the inflamed microenvironment. Gal-3 forms complexes when engaging soluble 4-1BB (s4-1BB), such complexes can be found in plasma from healthy controls (HC). However, patients with RA have markedly reduced levels of these circulating complexes. Thus, Gal-3 binding to 4-1BB facilitates complex depletion leading to reassembly of s4-1BB to the cell surface, acting as a decoy and resulting in shielding the membrane bound 4-1BB (mem4-1BB) from signaling.

## Results

### Galectin-3 Is a Binding Partner of 4-1BB

**Figure 1:**
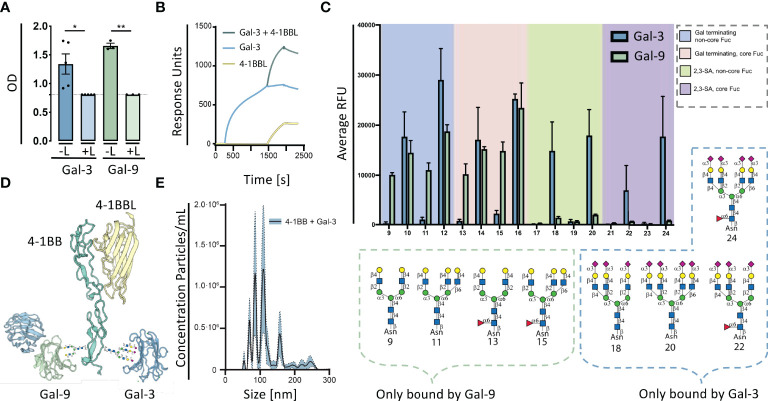
Galectins are capable of glycan dependent binding to 4-1BB. **(A)** 4-1BB binding to galectin-3 (Blue) or galectin-9 (Green) and with added 0.1 M Beta-lactose (L) measured by ELISA. Data are expressed as mean ± SEM (n=5 and n=3, respectively), Student’s paired t-test, *P<0.05, **P<0.01. **(B)** Surface plasmon resonance analysis of the binding of 4-1BBL (yellow line), Gal-3 (blue line), and co-injection hereof (green line), flowing along a BIAcore CM5 sensor chip with immobilized 4-1BB : Fc. **(C)** Glycan microarray analyses of human Galectin-9 using the N-Glycan array, Gal-9, was tested at 50 mg/mL. Binding with glycans 9 to 24 is depicted, the complete set of printed glycans and structural information can be found in **Table EV3**. Gal-9 binding is shown by green bars representing the mean and the error bars represent the SD among the values of four replicate spots. Reported Gal-3 binding is marked by blue bars again as mean with SD. The probes are grouped as indicated in the colored panels. Glycan structures bound specifically by Galectin-3 and Galectin-9 are displayed. **(D)** Proteins were modelled using SWISS-MODEL licensed under the CC BY-SA 4.0 Creative Commons Attribution-ShareAlike 4.0 International License modified with glycans in the CRD4 part of 4-1BB. Only the carbohydrate recognition domains of Gal-3 and Gal-9 are shown. **(E)** Detection of complexes generated by rh4-1BB and rhGal-3 (both 5 μg/ml) measured by nanoparticle tracking analyses. Data expressed in a scatter-mode by 5 recordings of 60s (n=5). Dashed line represents intraassay variation. The curve displayed are the difference between nanoparticle tracking analyses data obtained by rh4-1BB+rhGal-3 subtracted the sum of nanoparticle tracking analyses data obtained by the two individual proteins.

We examined 5 commonly expressed galectins (Gal-1,-2,-3,-8 and -9) associated with immune mediated inflammation and their ability to bind to 4-1BB by fluorescent anisotropy (**Table EV1**). Gal-3 and Gal-9N showed the strongest affinity towards 4-1BB, with K_D_-values between 1 and 2 μM (**Table EV1**). We confirmed that these interactions required functional CRDs, as binding could be blocked by lactose (**Figure 1A**) and for Gal-3, a mutant (Gal-3 R186S), with severely reduced affinity for endogenous glycans, did not bind 4-1BB (**Table EV1**). Gal-1, -2 and -8N were all capable of binding 4-1BB, but with a lower affinity. Because we used a 4-1BB : Fc construct, we also tested the Fc part of the construct as a control, which did not bind any of the galectins (**Table EV1**).

### Galectin-3 Competes With Neither 4-1BBL Nor Galectin-9 for the Binding to 4-1BB

The ability of 4-1BB to co-bind Gal-3 and 4-1BBL was examined by ELISA and surface plasmon resonance (SPR) analyses. The results demonstrate that 4-1BBL binds 4-1BB, as expected, but that binding of Gal-3 to 4-1BB does not compete for binding by 4-1BBL (**Figure 1B**). As Gal-3 and both terminal domains of Gal-9 are capable of binding 4-1BB, we examined if the two galectins interfered with each other’s binding to 4-1BB. Gal-3 and the N- and C-terminal domain of Gal-9 both exhibited a binding affinity to 4-1BB comparable with the fluorescent anisotropy data (**Table EV1**). Addition of either Gal-9C or Gal-9N to 4-1BB enhanced the subsequent binding of Gal-3 to a degree that surpassed the sum of the individual proteins (**Figure EV1**). This indicates a synergistic effect between these two galectins and 4-1BB in which binding of one increases the binding of the other. Collectively, Gal-9 binding to 4-1BB enhanced and stabilized the subsequent binding of Gal-3.

### Galectin-3 and Galectin-9 Have Different N-glycan Structural Binding Preferences

To define the potential differential binding of Gal-3 and Gal-9 to N-glycans, which is common types of 4-1BB modifications, we analyzed the binding of Gal-9 to a selection of 32 complex-type naturally occurring N-glycans using an N-Glycan array, on which Gal-3 was previously analyzed (28). The tetra-antennary N-glycan with four LacNAc (Gal-GlcNAc) sequences showed the strongest binding with Gal-9. By contrast, Gal-9 did not bind N-glycans with either α2,3- nor α2,6-linked sialic acid, known to bind Gal-3 (28). Gal-9 binding was observed toward biantennary and the 2,2,6-form triantennary structures, which were not bound by Gal-3 (**Figure 1C**). Gal-9 was also capable of binding a small tetrasaccharide LSTc, a 2,6-sialylated glycan that does not occur on N-glycan backbones. Taken together, although Gal-3 and Gal-9 have overlapping glycan ligands, they also have distinct separate structural preferences, making it possible for Gal-3 and Gal-9 to co-bind glycoproteins. In this vein, we modeled the protein structures of 4-1BB binding to 4-1BBL, Gal-3 and Gal-9, generated by SWISS-MODEL and superimposed N-glycans in the CRD4 region of 4-1BB (**Figure 1D**). The model depicts the potential of these two galectins to simultaneously bind 4-1BB.

Because Gal-3 is known to oligomerize after encountering a binding-partner (29), we analyzed whether the interaction between 4-1BB and Gal-3 was capable of forming larger complexes by the use of nanoparticle tracking analysis (NTA). In scatter detection mode (SDM), the NTA revealed the presence of large complexes and their size distribution. Distinct concentration peaks between 50 and 300 nm in hydrodynamic radius (R_h_) was detected (**Figure 1E**). The size separation between the concentration peaks had a relatively fixed increment of 20 nm, and major sizes of 90 and 110 nm in R_h_. Gal-3 did not oligomerise to form complexes without 4-1BB present (**Figure EV2**). With these data, we focused on the interactions between 4-1BB and Gal-3 and the implications of complex formation.

### Galectin-3 and 4-1BB Are Upregulated at the Site of Pathology in RA

**Figure 2:**
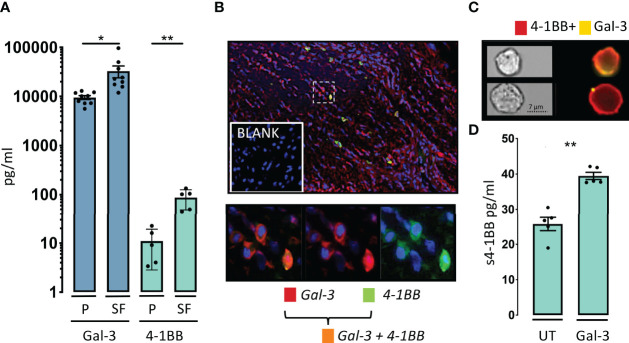
Gal-3 and 4-1BB are increased in the rheumatoid inflamed microenvironment. **(A)** In patients with rheumatoid arthritis (RA), soluble levels of Gal-3 (blue, n = 9) and soluble levels of 4-1BB (green, n = 5), were both increased in SF compared with P. Data are expressed as mean ± SEM, Student’s paired t-test, *P < 0.05, **P < 0.01, Bars represent mean (SEM). **(B)** Gal-3 (red) and 4-1BB (cyan) were co-expressed (orange) in the RA synovial membrane often localized to the sublining layer of the membrane (n = 3). Cells were nuclear stained by DAPI (blue), imaged obtained at x40. **(C)** Co-expression of 4-1BB (red) and Gal-3 (yellow) was also seen in the surface of CD3 and CD28 activated human CD4 T-cells by ImageStream. In the membrane of some 4-1BB+CD4 T-cells Gal-3 were found only in very distinct condense areas in others more diffuse n = 4. **(D)** Cultured CD4 T-cells were activated by CD3 and CD28 antibodies, with or without, added Gal-3 (400ng/ml), overnight (n = 5). Addition of Gal-3 resulted in a significantly increase in soluble (s) 4-1BB. Data expressed as mean ± SEM, student’s paired t-test, **P<0.01, Bars represent mean (SEM).

We continued by examining whether Gal-3 was elevated and co-expressed with 4-1BB at a site of inflammation in patients with RA, as a prototypic immune mediated inflammatory disease. In RA synovial fluid, soluble Gal-3 was elevated by 4-fold and s4-1BB by 12-fold compared with paired plasma control samples (**Figure 2A**). In the RA synovial membrane, co-expression of 4-1BB and Gal-3 was predominantly observed in the sub-lining layer of the inflamed synovial tissue (**Figure 2B**). Co-expression of 4-1BB and Gal-3 was also detected at the surface of purified CD3/CD28-activated human CD4 T cells (**Figure 2C**). We further examined whether addition of Gal-3 changed the membrane distribution of 4-1BB in these cells, which was found not to be the case (**Figure EV3**). Addition of Gal-3 to activated human CD4 T cells increased their shedding of 4-1BB (**Figure 2D**).

### Gal-3 and 4-1BB Interaction Is Involved in Complex-Depletion From Human Plasma

**Figure 3:**
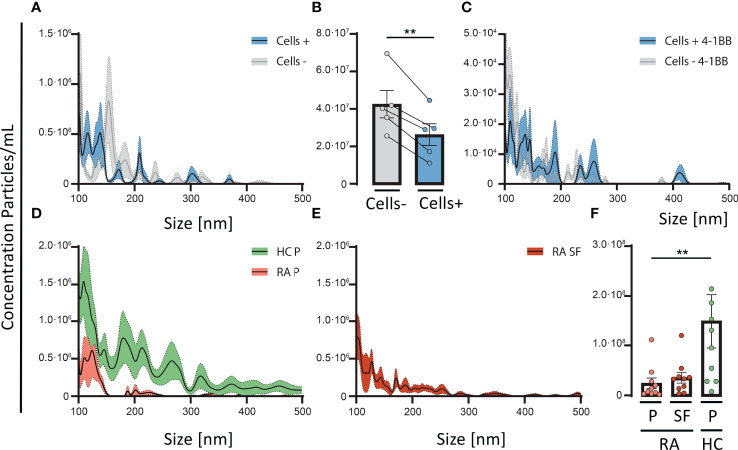
4-1BB positive T cells are involved in the depletion of soluble Gal-3 complexes. Normal human plasma was incubated with, or without, CD3/CD28-activated CD4 T-cells for 30 minutes at 37°C, followed by detection of Gal-3 positive particles measured by nanoparticle tracking analyses. **(A)** Activated CD4 T-cells (n = 5) resulted in a depletion of Gal-3 positive particles, especially with a diameter < 200nm. **(B)** CD3/CD28-activated CD4 T-cells (n = 5) incubated with plasma from healthy controls (HC) resulted in a significant reduction of Gal-3 positive particles compared with plasma not encountering activated T cells. **(A, B)** Data are expressed as mean ± SEM, Student’s paired t-test, **P < 0.01. **(C)** In a similar setup, plasma from HC were incubated for 30 minutes at 37°C, with 4-1BB transfected HEK293 cells (4-1BB^+^HEK293) and wild type (HEK293) (n = 5, HC plasma samples). 4-1BB^+^HEK293 cells depleted Gal-3 particles from plasma, more than wild type cells, primarily of complexes < 200 nm in size. **(D)** Comparing the distribution of Gal-3 positive particles in plasma from HC (Green, n = 10) and rheumatoid arthritis (Red, n = 10) the number of Gal-3 particles were decreased in RA plasma. **(E)** RA synovial fluid (n = 10) showed a similar pattern of Gal-3 positive particles as seen in RA plasma. **(F)** The total number of Gal-3 complexes were significantly decreased in RA plasma compared with HC plasma, **P < 0.01 (unpaired t-test). Data are expressed as mean ± SEM, unpaired Student’s t-test.

Since Gal-3 can form complexes, with 4-1BB, we examined if these complexes were present *in vivo* and furthermore if activated T-cells and 4-1BB^+^HEK293 cells would influence the concentration of soluble Gal-3 complexes in solution. In normal human plasma, we detected Gal-3 containing complexes with a size of > 100 nm, suggesting that Gal-3 engages in complex formation with glycoproteins (**Figure 3A**). Addition of activated CD4+ T cells to normal human plasma led to a 1.6-fold decrease in these Gal-3 complexes (**Figure 3A, B**).

Furthermore, addition of transfected HEK293 cells expressing membrane bound 4-1BB, lead to a higher decrease of Gal-3 complexes from plasma than WT HEK293 cells, suggesting a role of 4-1BB (**Figure 3C**). Both activated T cells, and 4-1BB^+^HEK293 cells led to a similar change in the distribution pattern of Gal-3 complexes in plasma with ablation of complexes primarily between 100-200 nm in size (**Figure 3A, C**).

Since activated T-cells and 4-1BB expressing HEK293 cells could deplete Gal-3 complexes from plasma, we explored whether plasma from RA patients exhibit signs of Gal-3 complex depletion. In RA, the concentration of complexes containing Gal-3 was significantly reduced. Gal-3 complexes in RA plasma were reduced by about 4-fold (**Figure 3D, E**) and in paired synovial fluid by about 3-fold (**Figure 3F**), compared with plasma from HC (*P<0.05, P=0.05, respectively). The concentration of complexes did neither correlate with the total sGal-3 protein levels in synovial fluid nor in plasma from RA (**Figure EV4**).

### Functional Implications of 4-1BB/Gal-3 Complex Depletion

**Figure 4:**
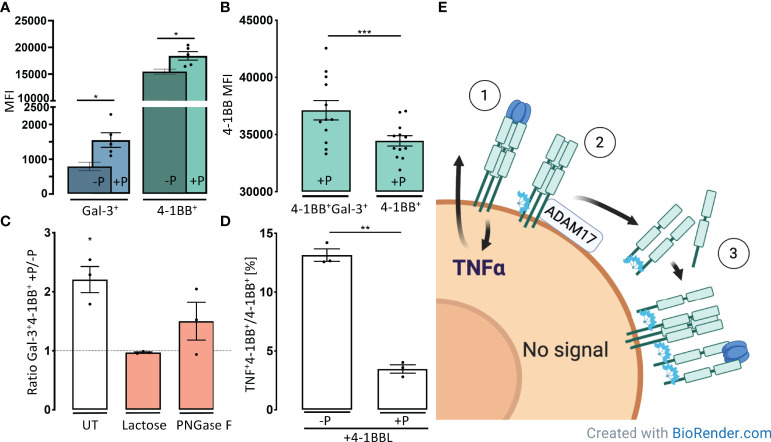
Gal-3 forms complexes with s4-1BB that can attach to the cell surface and shield cells from 4-1BBL induction of TNFα. **(A)** CD3/CD28-activated CD4 T-cells were incubated for 30 minutes at 37°C with (+P), or without, plasma (-P) from healthy controls (HC). Cells were then examined by flow cytometry for the membrane median fluorescence intensity (MFI) of Gal-3 measured on 4-1BB+Gal-3+ CD4+ T cells (Blue) and MFI of 4-1BB measured on 4-1BB+ CD4+ T cells (Green). Bars represent mean (SEM) as ratio of MFI on cells incubated +/- plasma (n = 5, *P < 0.05). **(B)** Expression of 4-1BB on 4-1BB transfected HEK293 cells after incubation with plasma (+P) from healthy controls. Cells that were doublet positive for 4-1BB and Gal-3 showed a significant higher 4-1BB MFI compared with single positives (n = 12). Data are expressed as mean ± SEM, Student’s paired t-test, ***P < 0.001. **(C)** The proportion of Gal-3^+^ and 4-1BB^+^ T cells were increased after 30 minutes incubation in HC plasma. The proportion of the double positive Gal-3+4-1BB+ cells decreased by addition of Lactose or PNGase F, compared with untreated (UT). Bars represent mean (SEM) as ratio of Gal-3+4-1BB+ cells with or without HC plasma (n = 3, *P < 0.05). **(D)** Activated CD4^+^, 4-1BB^+^ T cells incubated with or without, plasma from healthy controls and subsequently stimulated with 4-1BBL. Plasma preincubation reduced CD4 T-cells ability to produce TNFα upon 4-1BBL stimulation (n = 3). Bars represent mean (SEM) as ratio of TNFα producing 4-1BB+ CD4 T cells out of all 4-1BB+ CD4 T cells **P < 0.01. **(E)** Based on our results, we suggest a model where 4-1BBL induce TNFα synthesis ① and Gal-3 increases shedding of 4-1BB ②. Gal-3 mediates complex formation engaging with s4-1BB. These 4-1BB/Gal-3 complexes reassemble on the cell surface by binding to membrane expressed 4-1BB ③, creating a decoy mechanism that blocks for further 4-1BBL stimulation.

We continued by examine the cellular expression of Gal-3 and 4-1BB on activated CD4^+^ T-cells after incubation with plasma from healthy controls. After co-incubation with plasma, only the 4-1BB^+^ fraction of CD4^+^ T-cells showed a 2-fold increase in Gal-3 MFI and in addition, these cells also increased the surface expression of 4-1BB MFI (**Figure 4A**).

Furthermore, after co-incubation and complex depletion from plasma the 4-1BB MFI also increased significantly on HEK293 cells co-expressing both Gal-3+ and 4-1BB+ compared with HEK293 cells only expressing 4-1BB (**Figure 4B**, **EV5**). Moreover, the fraction of Gal-3+4-1BB^high^ cells was reduced to levels of non-plasma treated cells after treatment with lactose or by PNGase pre-treatment of the cells to decrease N-glycans interacting with Gal-3 (**Figure 4C**). These results indicate that the large, soluble 4-1BB/Gal-3 complexes from plasma can attach to the cell surface of 4-1BB expressing cells.

### Soluble Complexes of Gal-3 and 4-1BB Inhibits Cytokine Production

We examined whether these soluble complexes containing both Gal-3 and 4-1BB could bind to the cell surface and interfere with 4-1BB signaling. After pre-incubation activated CD4 T-cells with plasma, the fraction of 4-1BB+CD4+ T cells that produced TNFα was reduced by 3.8-fold upon 4-1BBL stimulation (P < 0.01) (**Figure 4D**, **EV5**). We continued to examine if this process was influenced by a highly glycosylated environment, observed in the inflamed joint, with its high amount of exposed glycosylated extracellular matrix (ECM) proteins and immunoglobulins. Gal-3 were capable of binding both laminin and fibronectin, but not collagens, IgG and IgM (**Figure EV6**).

Since Gal-3 strongly bound to laminin, we evaluated if the 4-1BB/Gal-3 complexes could be removed from the surface of activated CD4+ T cells by encountering a laminin coated surface. Furthermore, the 4-1BBL mediated TNFα production in CD4+ T-cells was not influenced after these had encountered a laminin coated surface prior to 4-1BBL co-incubation (**Figure EV7**).

Our observations suggest that Gal-3 increases shedding of 4-1BB which subsequently leads to formation of large soluble complexes between s4-1BB and Gal-3. These 4-1BB/Gal-3 complexes reassemble on the cell surface by binding to mem4-1BB. This complex deposition onto the membrane of these 4-1BB+ CD4+ T cells limits further 4-1BBL stimulation (**Figure 4E**).

## Discussion

The present study provides novel insight into the posttranslational regulation of the 4-1BB receptor, which is N-glycosylated, mediating its ability to bind Gal-3 and hereby promotes oligomerization. As a novel point, we observed that Gal-3 can bind both the soluble and the membranous form of 4-1BB, leading to inhibition of 4-1BBL stimulation. This finding helps to explain the complicated nature of the 4-1BB signalosome. Our results support the possibility that multiple galectins are capable of binding to the glycosylated 4-1BB receptor, but that Gal-3 and Gal-9 (N/C) binds to 4-1BB with the highest affinity.

The binding of Gal-3 does not block the co-binding of 4-1BBL, but mediates increased shedding of mem4-1BB. The predicted binding sites of Gal-3 to 4-1BB is through its N-glycans located in the cysteine rich domain 4 of human 4-1BB at Asn-138 and Asn-149 (30, 31). Both amino acids are >35 amino acids above the predicted ADAM-17 cleavage site located only two amino acids from the transmembrane region (16). Thus, shed 4-1BB retains its glycosylation, enabling both Gal-3 and Gal-9 binding. Further, non-competitive binding of Gal-3 and Gal-9 to 4-1BB is possible since both galectins have separate structural preferences to complex N-glycans in large determined by branching and sialyation. While Gal-9 binding to 4-1BB increases the TNFα production (16), Gal-3 oppositely seems to hinder TNFα production mediated by 4-1BBL. This mechanism involves a series of events initiated by mem4-1BB expression, then followed by shedding by ADAM17. In turn, secretion of Gal-3 and the assembly of the 4-1BB/Gal-3 leads to the formation of large complexes. The reassembly of 4-1BB/Gal-3 complexes on the cell surface is mediated through binding between Gal-3 and mem4-1BB. An attractive explanation for the enhanced binding by these large complexes derives from the high avidity resulting from the polyvalent binding of the 4-1BB/Gal-3 complexes to the membrane-tethered form of 4-1BB (32). This phenomenon is also experimentally demonstrated for polyvalent lectin complexes (33). The shielding of mem4-1BB could ultimately lead to a situation whereby 4-1BBL can no longer induce cytokine production. Further, Gal-3 can bind to laminin and fibronectin as also previously demonstrated (34). Laminin and fibronectin are glycosylated ECM proteins present in the inflamed tissue, including the arthritic joint (35). However, we detected no influence on the complex mediated shielding of mem4-1BB after the cells encountered laminin. Thus, it is unlikely that the expression of laminin in the local inflamed environment alters mem4-1BB shielding after assembly of 4-1BB/Gal-3 complexes onto the cell surface.

Thus, Gal-3 and Gal-9 have opposite impacts on the ability of 4-1BBL to induce a 4-1BB signal, indicating that the function of 4-1BB^+^ T cells, is regulated by the balance of galectins in the local microenvironment.

We observed a near-absence of soluble 4-1BB/Gal-3 complexes in the synovial fluid of RA patients. Although much points to the binding of the complexes to activated T cells, we cannot rule out that this absence is additionally affected by local factors that influence glycosylation, or through depletion of Gal-3 complexes due to binding between Gal-3 and extracellular matrix proteins (36, 37). Also, our study does not take into account that glycosylation is modified by the inflammatory environment (38), potentially affecting cytokine stability and receptor interactions (39). The ability of 4-1BB/Gal-3 complexes to bind to cell surfaces with an inhibition of 4-1BBL signaling, is somewhat in contrast to reports of agonistic antibodies towards 4-1BB ameliorating autoimmunity in a mouse model (40). This paradoxical effect was speculated both to be due to complement activation, as these antibodies are of the IgG2 subtype, as well as activation-induced cell death (41). Further, it is important to consider that mouse and human 4-1BB likely differ in their glycosylation, which could also influence the described mechanism (31). Although no studies have yet reported association between 4-1BB expression and reactivity to antibody treatments in human trials, it will likely follow the pattern of other checkpoint molecules, with only weak association to efficacy (42, 43).

We predict, based on the ability of Gal-3 to generate 4-1BB decoy receptor complexes, that 4-1BB will also be an inconsistent biomarker of agonistic 4-1BB efficacy. At the moment, urelumab and utomilumab are the two lead molecules specifically targeting 4-1BB in oncology. As the binding affinity between galectins and 4-1BB are ~50-60 times weaker than the two reported monoclonal antibodies, one may speculate that these could disrupt Gal-3s binding to 4-1BB (31). However, it has been reported that neither urelumab nor utomilumab interfere with Gal-9 binding to 4-1BB (15) (31). Further, Gal-3 and Gal-9 both bind to glycans on the cysteine-rich domain 4 part of 4-1BB, which is structurally separated from that of both agonistic antibodies. Additionally, since Gal-3 and Gal-9 have similar binding affinities, we do not expect antibody therapies to block co-binding of galectins that would modulate the resulting 4-1BB signaling. The formation of 4-1BB/Gal-3 complexes in the inflamed microenvironment, on the other hand, would likely block subsequent binding of targeted antibodies to the mem4-1BB. This points to a key role of Gal-3 levels for 4-1BB signaling in the local inflamed environment. Indeed, the high-avidity binding supported by the polyvalent interaction also suggest that such complexes may be stable even in the presence of the antibodies. Our results are also supported by previous studies showing that in the tumor microenvironment Gal-3 causes predominantly reduced T cell activation to some degree reversible by Gal-3 blocking (27, 44, 45).

In the present study, we describe one member of the TNFRSF and its ability to engage in complex formation with Gal-3. We predict that other TNFRSF members will be subjected to a similar mechanism, as the TNFRSF shares signaling properties, glycosylation, receptor oligomerization and downstream signaling pathways (1, 6). Further, altered Gal-3 and Gal-9 expression is found in several inflammatory diseases (46) and in the tumor microenvironment of several cancers (27). A recent study links the effectiveness of checkpoint inhibitor-based immunotherapy to the local levels of Gal-3 (47). Collectively, the mechanistic insight generated from our studies clearly supports the possibility that the interaction observed may be relevant to other diseases and glycosylated immunoreceptors expressed by immune or stromal cells (46, 48).

In conclusion, Gal-3 binds 4-1BB without blocking for subsequently 4-1BBL binding, Gal-3 increased shedding of mem4-1BB, and mediated complex formation with s4-1BB. These soluble 4-1BB/Gal-3 complexes may assemble on the cell surface by binding to mem4-1BB, thus creating a decoy mechanism weakening 4-1BBL stimulation.

## Materials and Methods

### Study Participants

A cross-sectional, paired set of peripheral blood mononuclear cells (PBMCs) and SF mononuclear cells (SFMCs) were obtained from patients with chronic RA and at least one swollen joint (n = 20) at the outpatient clinic at Aarhus University Hospital at the time of therapeutic arthrocentesis (Table 1). Synovial tissues were obtained from joints at arthroplasty from 3 patients with chronic RA. Plasma and PBMC’s from healthy controls (HC) (n = 10), were obtained from the Danish Blood Bank, Aarhus University Hospital.

### Ethics

The studies were approved by the Regional Ethics Committee (2012-291-12) and the subjects’ written informed consents were obtained according to the Declaration of Helsinki.

### Cell Culture

The SFMCs cultured in RPMI medium supplemented with 10% fetal calf serum (FCS), penicillin, streptomycin, and glutamine at a density of 2x10^6^ cells/ml. For the stimulation experiments, a combination of recombinant human (rh)4-1BB.Fc chimera (100ng/ml) (cat. no. 838-4B) and rh4-1BBL (100ng/ml), rhGal-3 (400ng/ml) (R&D Systems, USA). The cells were cultured as previously described (16). Transfected human embryonic kidney 293 (HEK293) cells continually expressing 4-1BB were established using the FlpIn system (Life Technologies) as described (16). 4-1BB+HEK293 cells were incubated with either 0.1 M Lactose (Sigma-Aldrich) or PNGase F 100 μg/ml (R&D Systems, USA) prior to plasma addition, before processed for flow cytometry.

### Activation of Activated Human CD4 T Cells

CD4+ T cells were isolated by negative selection from HC PBMCs (n=6) by a Human CD4+ T cell isolation kit following the instructions of the manufacturer (Stemcell). The T cells were subsequently stimulated with anti-CD3, anti-CD28 (BD Pharmingen) and IL-2 (Sigma-Aldrich) as previously described (49). The activated CD4+ T cells were used in two set-ups A. cultured with or without rhGal-9 (400ng/ml), rhGal-3 (400ng/ml) (R&D Systems, USA) analysis by ImageStream. B. Cells were cultured with, or without, 20% plasma from HCs (n=5) for 30 min. For the intracellular CD4 T cell analyses, cells were cultured under serum free conditions and activated with anti-CD3, anti-CD28 (BD Pharmingen) for 16 hours. Cells were then washed and media was added containing 20% HC plasma and Brefeldin A (10 μg/ml) for 30 min before cells were cultured on either a laminin coated (2 ug/ml) or uncoated surface for 30 min. Hereafter the CD4+ T cells were stimulated with rh4-1BBL (400ng/ml) for 4 hours. The samples were directly processed for further NanoSight, Flow cytometry or ImageStream analyses.

### ELISA for 4-1BB

Soluble 4-1BB levels were measured as earlier publish (18). The galectin+4-1BB-binding assay were coated with either rhGal-3 or rhGal-9 (R&D Systems, USA) at 1 μg/ml with, or without, 0.1 M Beta-lactose (Sigma, L3750-100G) followed by addition of rh4-1BB 1 μg/ml (R&D Systems, USA). Finally, the inhibition-binding assays were evaluated by adding a biotinylated anti-4-1BB antibody 0.5 μg/ml (R&D Systems, USA) followed by Streptavidin HRP. The inhibition assays were incubating O.N at 4°C.

### Flow Cytometry and ImageStream Analyses

CD4+ T cells were surface stained for 30 min using the following murine monoclonal antibodies: APC-anti 4-1BB (Cat. 309810), PE-anti Gal-3 (Cat. 126706), FITC-anti CD3 (BioLegend) and LIVE/DEAD (Life Technologies). Intracellularly staining were preceded by blocking with 50 µg/ml mouse IgG for 15 min before staining with an anti-TNFα antibody (Franklin Lakes, USA). Cells were washed, fixed and analyzed on a NovoCyte Quanteon™ or Amnis^®^ ImageStream^®^ Imagine Flow Cytometer. Spectral overlap was compensated using antibody-coated beads (eBioscience). Gating was done on live cells using fluorescence minus one (FMO). Data were analyzed using IDEAS for windows version 6.2 and/or FlowJo for Mac software version 10.1.

Imagestream gating included cells in focus, single cells and CD3 positive cells. A membrane mask was defined based on the CD3 stain using morphology mask and erode. Membrane mask included the outer three pixels. Within this mask the aggregation of 4-1BB was calculated and the surface specific MFI values determined.

### Nanoparticle Tracking Analyses of Gal-3 Complexes

Quantum dot antibody coupling was done using SiteClick™ Qdot™ 655 Antibody Labeling Kit (Molecular Probes, S10453) according to manufactures instructions. The conjugation can be summarized as follows: anti-Gal-3 monoclonal antibody (Thermo Fischer #A3A12) was concentrated in antibody preparation buffer to ensure a concentration of 2mg/ml or above. Next, antibody carbohydrate domain was modified by incubation with β-galactosidase for 4 hours at 37°C. Azide modification was achieved through incubation with UDP-GalT enzyme overnight at 30°C. Carbohydrate modified antibody was purified and concentrated through a series of centrifugation steps using a membrane concentrator and buffer is changed to Tris pH 7.0. Finally, DIBO modified quantum dot nanocrystals were attached overnight at 25°C and labelled antibody was stored at 4°C.

NTA was performed using a NanoSight NS300 system (Malvern Panalytical). System was configured with a 405-nm laser, a high-sensitivity scientific complementary metal–oxide–semiconductor camera (OrcaFlash2.8, Hamamatsu C11440; Malvern Panalytical), a syringe pump and for fluorescence detection mode (FDM) a 650 nm long pass filter. For samples analyzed in SDM such as recombinant proteins, the sample chamber was washed twice before each measurement. Samples were diluted to a concentration of 5 µg/ml and thoroughly mixed before injection into the sample chamber using 1-ml syringes. Recordings were made with temperature control fixed at 23°C. Recordings were captured continuously during a steady flow at flowrate 10 μL/min with 5 recordings of 60s duration separated by a 5s lag time in between each recording. Videos were collected and analyzed using NanoSight software (version 3.3 and 3.4 with concentration upgrade). Automatic settings were used for minimal expected particle size, minimum track length, and blur setting. SDM Camera level (CL) 13 and detection threshold (DT) 5 was kept constant for all samples to be directly compared. FDM CL was set to maximum (level 16) and DT was set close to minimum (level 3). Sample buffer was PBS 1 mM EDTA. Human plasma samples were analyzed at a 1:10 dilution in PBS 1 mM EDTA with a 1:20,000 dilution of specific antibody conjugates. A 100nm cut-off was established for all samples including Quantum dot coupled antibodies to exclude free quantum dot conjugates.

### Fluorescent Anisotropy Assay

Recombinant human galectins were produced as previously described (50). A fluorescence anisotropy (FA) assay was used to determine the affinity of (rh)4-1BB Fc chimera protein (R&D Systems, USA) or (rh)IgG1 Fc protein (R&D Systems, USA) to a panel of (rh)galectins in solution, as described previously (51, 52). Fixed concentrations of 0.3, 1.2, 0.2, 0.3, 0.2, 0.6, and 0.6 µM was used for (rh)galectin-1 (C3S), -2, -3, -8C, -8N, -9C, and -9N, respectively, and fluorescent probes were as previously described (51). For (rh)galectin-3 (R186S) a fixed concentration of 2.5 µM was used and the fluorescent probe was as described before (52). Calculations of average K_D_-values was as previously described (51).

### Surface Plasmon Resonance

Surface plasmon resonance analysis was performed using a Biacore 3000 instrument (Biacore, Uppsala, Sweden). The Biacore sensor chip (type CM5) was activated with a 1:2 mixture of 0.2 M N-ethyl-N’-(3- dimethylaminopropyl) carbodiimide and 0.05 M N-hydroxysuccimide in water. Next, rh4-1BB.Fc (cat. no. 838-4B; R&D Systems) and rhGal-3 (E. Coli produced as described (50)) was immobilized in 10 mM sodium acetate (pH 4.0) and the remaining binding sites were blocked with 1 M ethanolamine (pH 8.5). The resulting density was approximately 90fmol protein/mm^2^. Sensorgrams were generated using protein at 50 nM and CaHBS with 2 mM free Ca2Cl2 (10 mM Hepes, 150 mM NaCl, 1.5 mM CaCl2, 1.0 mM EGTA, +0.005% P20, pH 7.4) as running buffer (16). The analyses were performed with the following recombinant human proteins: 4-1BBL (50 mM), Gal-3 (50mM), 4-1BB : Fc (3-200 nM), Gal-9C (200 nM), and Gal-9N (200nM).

### Immunofluorescence of RA Synovial Tissue

Paraffin embedded RA synovial tissue slides were deparaffinized and subjected to antigen retrieval. Non-specific binding was blocked by incubating in PBS with 0.5% BSA and 10% donkey serum for 30 minutes at RT and avidin and biotin block (Dako, Denmark) for 10 min. Slides were stained using a combination of biotinylated mice anti-4-1BB, unconjugated rabbit anti-Gal-3, or anti-Gal-9 antibodies (All from ThermoFisher) followed by streptavidin Alexa 546 and donkey anti-rabbit Alexa 647 (Jackson ImmunoResearch).

### Microarray Analysis

Extracellular matrix microarray; The glycosylated extracellular matrix proteins (ECM) were printed on Oncyte^®^ nitrocellulose film slides (Grace Bio-Labs) using a sciFLEXARRAYER S11 (Scienion). Collagen I (234138, Sigma-Aldrich), Elastin (324751, Sigma-Aldrich), Collagen II (CC052, Sigma-Aldrich), Laminin (AG56P, Sigma-Aldrich), Thrombospondin (605225, Sigma-Aldrich), Fibronectin (341635, Sigma-Aldrich), Collagen IV (CC076, Sigma-Aldrich), Collagen III (CC054, Sigma-Aldrich), Vitronectin (CC080, Sigma-Aldrich), IgM bulk, IgG bulk, IgG FC (Sigma-Aldrich), and Chicken collagen II (C9301, Sigma-Aldrich) were purchased from various vendors mentioned. Collagen II was solubilized by digestion in 0.25% acetic acid (pH 3.1) over several hours at 2-8°C, with occasional vortexing and sonicating. All proteins were then dissolved in PBS, printed at a concentration of 100 μg/ml in replicates of 4 spots/protein. After printing, the slides were incubated overnight at 4°C in a cold room. The next day, the slides were treated using Super G Plus™ Protein Preservative (Grace Bio-Labs) as per manufacturers recommendation to block the slides from non-specific binding and storage. The slides were stored at -20°C in an airtight tube container until use. The printed proteins were verified independently with corresponding antibodies against them.

For the assay with Gal-3, after rehydration using TSM buffer (20 mM Tris–HCl, 150 mM sodium chloride, 0.2 mM calcium chloride, and 0.2 mM magnesium chloride), the microarray slides were probed with or without 20 μg/ml biotinylated rhGal-3 [Laboratory stock (52)]. The bound Gal-3 were detected with cyanine 5-streptavidin at 1 μg/mL (Invitrogen). After incubation, the binding signals were directly quantified. Slides were scanned with a Genepix 4300A microarray scanner (Molecular Devices, Sunnyvale, CA), photomultiplier (PMT): 450, Laser Power (LP): 25).

The N-glycan microarray construction was as described previously (28). After rehydration using TSM buffer (20 mM Tris–HCl, 150 mM sodium chloride, 0.2 mM calcium chloride, and 0.2 mM magnesium chloride), the microarray slides were probed with recombinant human Gal-9 (50 μg/ml) (9064-GA, R&D Systems) at room temperature for 1 h. After washing with TSM washing buffer (TSM buffer with 0.05% Tween-20), the slides were incubated with anti-human Gal-9 (50 μg/ml) at room temperature for 1 h. In both array setups the bound anti-Gal-9 was detected with Alexa Fluor 633-labelled anti-goat IgG (H+L) at 5 μg/ml (A21086, Thermo Fischer). After incubation, the binding signals were directly quantified. Slides were scanned with a Genepix 4300A, microarray scanner from Molecular Devices (Sunnyvale, CA).

Regarding both microarrays spot-based signal intensities were quantified using GenePix Pro 7 (Molecular Devices). The raw data from the software was further processed using Microsoft Excel to obtain the background subtracted mean relative fluorescence intensity for the four replicates of each glycan along with the standard deviation.

### Statistics

Statistical analyses and graphs were done using GraphPad Prism 7 for Mac (GraphPad Software). Normally distributed data are represented as mean ± SEM and were analyzed by Student’s paired t-test if not otherwise stated. A two-sided *P* value < 0.05 was considered statistically significant.

## Data Availability Statement

The raw data supporting the conclusions of this article will be made available by the authors, without undue reservation.

## Ethics Statement

The studies involving human participants were reviewed and approved by The Regional Ethics Committee. The patients/participants provided their written informed consent to participate in this study.

## Author Contributions

MN conceived the project, helped to collect the samples, performed experiments, analyzed data, and wrote the manuscript. JS, TK, CG, AM, SG, and KJ-M helped to collect the samples, performed experiments and analyzed data. TW, HL and BD conceived the project, supervised the work, analyzed data, and co-wrote the manuscript. All authors discussed the results and commented on the manuscript.

## Funding

KJ-M and TV-J kindly acknowledge a generous grant from Aarhus University Research Foundation (NOVA, AUFF-E-2015-FLS-9-6). MN was also supported by a grant from Aarhus University Research Foundation (NOVA, AUFF-E-2016-9-27) and the Danish Rheumatoid Association (R188-A6589). We acknowledge the resources and training of the Microarray Resource of the National Center for Functional Glycomics (NCFG) at Beth Israel Deaconess Medical Center, Harvard Medical School (supporting grants P41GM103694 and R24GM137763).

## Conflict of Interest

The authors declare that the research was conducted in the absence of any commercial or financial relationships that could be construed as a potential conflict of interest.

## Publisher’s Note

All claims expressed in this article are solely those of the authors and do not necessarily represent those of their affiliated organizations, or those of the publisher, the editors and the reviewers. Any product that may be evaluated in this article, or claim that may be made by its manufacturer, is not guaranteed or endorsed by the publisher.

## Supplementary Material

Supplementary Figure 1 Co-binding of 4-1BB (cyan) and the Gal-9C (Green) and Gal-9N (Green) terminal to immobilized Gal-3. Binding of Gal-9N and C to 4-1BB enhanced the subsequent binding to Gal-3 and also the stability of the interaction. At fixed concentrations, Gal-9N showed a stronger binding to 4-1BB compared to Gal-9C.

Supplementary Figure 2
**(A)** Detection of complexes generated by rhGal-3 (5 μg/ml), **(B)** rh4-1BB (5 μg/ml) and **(C)** a combination hereof, measured by nanoparticle tracking analyses. Data obtained in scatter-mode by 5 separate recordings of 60s. Dashed line represents intraassay variation. The differences between these curves and the combined curve are depicted in **Figure 1E**.

Supplementary Figure 3
**(A)** CD3/CD28-activated CD4 T-cells were gated on ImageStream and evaluated on their membrane distribution of 4-1BB, divided in 4-1BB aggregated or homogeneous distributed membrane 4-1BB on CD4+ T Cells. **(B)** Representative images of Gal-9 (Yellow) and 4-1BB (Red) co-expressing CD4+ T Cells. **(C)** Activated T cells were incubated for 30 minutes at 4°C with, or without, Gal-3 or Gal-9. Cells were then examined by ImageStream for their membrane distribution of 4-1BB. Ratio of aggregated (Grey) and homogeneous (Orange) with or without Gal-3 or Gal-9 (n=4). Upon Gal-9 stimulation significant more 4-1BB were homogeneous distributed in the membrane compared with untreated cells, P<0.05. **(D)** Activated CD4+ T Cells with homogeneously expressed membrane 4-1BB (Orange) had a significant higher 4-1BB MFI compared with cells on which 4-1BB was aggregated in the membrane (Grey), P < 0.0001.

Supplementary Figure 4 The soluble (s) levels of Gal-3 were measured by ELISA and the particle form of Gal-3 by nanoparticle tracking analyses in patients with RA (n=8) did not show any correlation. **(A)** Neither between sGal-3 levels in the synovial fluid (SF) patients and the concentration of complexes or particles, **(B)** nor between sGal-3 levels found in plasma (P) and the concentration of complexes measured also in plasma.

Supplementary Figure 5 Gating strategy for permeabilized human CD3/CD28-activated CD4 T cells intracellularly stained for TNFα. **(A)** Live, single cells were tested for co-expression of 4-1BB and TNFα, gates based on FMOs, evaluated with, or without, incubation in HC plasma for 30 min. **(B)** 4-1BB+ HEK293 cells were stained for Gal-3 expression after incubation in in HC plasma for 30 min. Live, single cells were evaluated for 4-1BB and Gal-3 expression, gates were again based on FMOs. Gal-3+ were only seen on 4-1BB+ HEK293 cells (Blue).

Supplementary Figure 6
**(A)** The glycosylated extracellular matrix proteins (ECM) were printed on microarray slides coated with nitrocellulose. Human Galectin-3 were tested with 20 μg/mL. The error bars (Black) represent the SD among the values of four replicate spots. Reported Gal-3 binding is marked by transparent bars. The probes are grouped as indicated in the colored panels. The binding signals were directly quantified and displayed as mean relative fluorescence intensity (RFU).

Supplementary Figure 7 Co-incubation of CD4+, 4-1BB+ T cells with plasma from healthy controls on a laminin coated or uncoated surface for 30 min. before being stimulated with or without 4-1BBL. Encountering a laminin coated surface did not led to increased TNFα production in the 4-1BB+ CD4+ T cells. Further laminin did not indirectly inhibit the effect of 4-1BBL stimulation on 4-1BB+ CD4+ T cells. **(A)** In the first and second bar, activated CD4^+^, 4-1BB^+^ T cells with plasma from healthy controls were cultured on a laminin coated or uncoated surface for 30 min. before being stimulated with 4-1BBL (n = 5). In the third bar, co-incubation of CD4+, 4-1BB+ T cells with plasma from healthy controls on a laminin coated surface with or without 4-1BBL stimulation (n = 4). Bars represent mean (SEM) as the fraction of TNFα producing 4-1BB+ CD4 T cells. Second bar represents a ratio of cells cultured with laminin compared to without laminin prior to 4-1BBL stimulation. Second bar represents a ratio of cells cultured with laminin but without 4-1BBL stimulation. *P < 0.05.

Supplementary Table 1 Average calculated KD-values (µM) for 4-1BB toward different galectins measured with fluorescence anisotropy. Two experimental replicates were used for calculating the average K_d_-values. Between 0.2 and 2.5 µM of each galectin with 0.02 µM of corresponding fluorescence probe was tested in the presence of a range of concentrations (0.07-4.75 µM) of (rh)4-1BB or (rh)IgG1 Fc; anisotropy was measured and average K_d_-values were calculated (in total 6-20 (rh)4-1BB concentration measuring points generating ≥ 15% inhibition were used to calculate the average K_d_-values). All galectins were tested against the same production batch of (rh)4-1BB or (rh)IgG1 Fc. NB; Not binding (*i.e.* < 15% inhibition or no inhibition at highest concentration tested). NT; Not tested. N/A; Not applicable.

Supplementary Table 2 Characteristics of included patients with rheumatoid arthritis (RA). Data are expressed as % or median (IQR). DAS28CRP, disease activity score 28 based on CRP; RA, rheumatoid arthritis. Seropositive: Presence of IgM-rheumatoid factor or anti-citrullinated protein antibodies. DAS28CRP: Disease Activity Score-28 for Rheumatoid Arthritis with CRP.

Supplementary Table 3 Related to **Figure 1C**. The raw data of the microarray analysis of galectin-3 and galectin-9 at 50 μg/ml in the N-glycan array, mean and SD among the values of four replicate spots. Gal-3 data were previously published (27). The structures specifically-bound by Gal-3 and Gal-9 are highlighted in Blue and Green, respectively.
