# Supplementary material for: Galectin-3 Decreases 4-1BBL Bioactivity by Crosslinking Soluble and Membrane Expressed 4-1BB
Source: Front Immunol. 2022 Jun 24;13:915890. doi: 10.3389/fimmu.2022.915890 (PMC9263355; doi:10.3389/fimmu.2022.915890)
Supplement: Supplementary Figure 1 — Co-binding of 4-1BB (cyan) and the Gal-9C (Green) and Gal-9N (Green) terminal to immobilized Gal-3. Binding of Gal-9N and C to 4-1BB enhanced the subsequent binding to Gal-3 and also the stability of the interaction. At fixed concentrations, Gal-9N showed a stronger binding to 4-1BB compared to Gal-9C. [file Presentation_1.pdf]

Supplemetary

Figure EV1

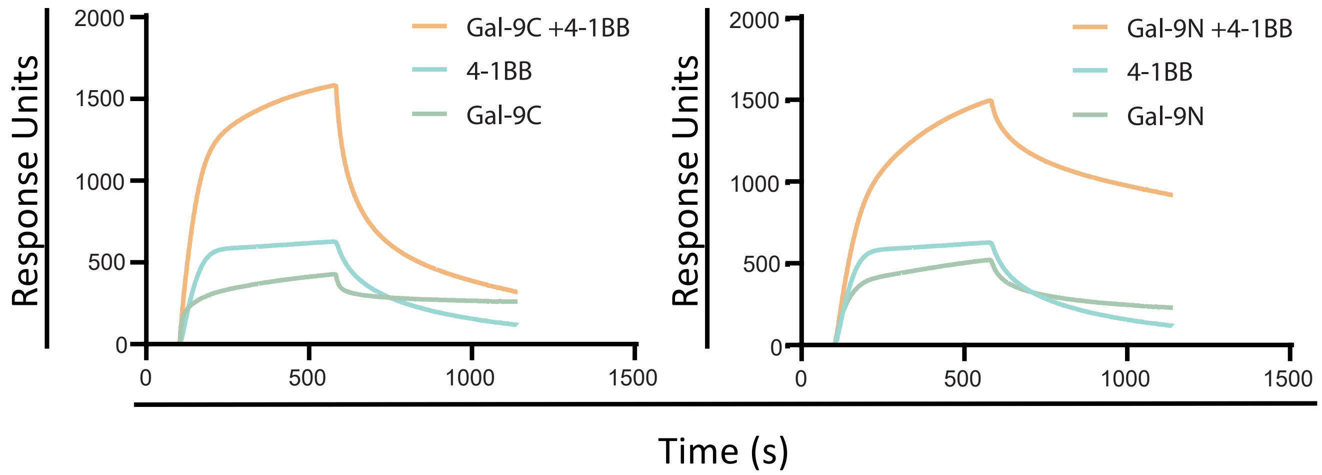
Response Units
2000
1500
1000
500
0
0
500
1000
1500
Gal-9C +4-1BB
4-1BB
Gal-9C
Response Units
2000
1500
1000
500
0
0
500
1000
1500
Gal-9N +4-1BB
4-1BB
Gal-9N
Time (s)

Figure EV2

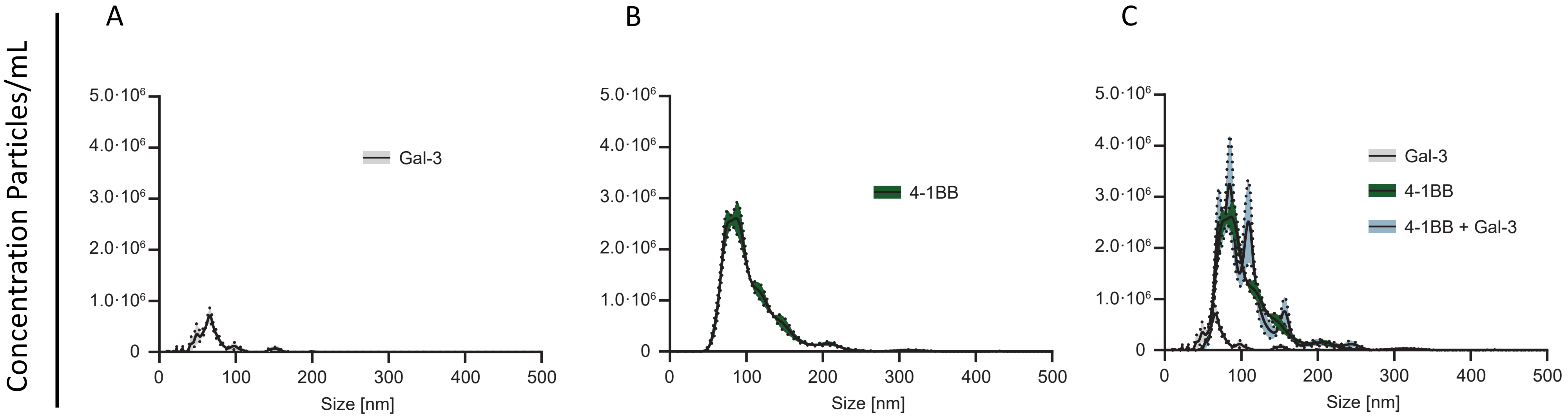

Concentration Particles/mL
A
5.0·10⁶
4.0·10⁶
3.0·10⁶
2.0·10⁶
1.0·10⁶
0
Gal-3
0
100
200
300
400
500
Size [nm]
B
4-1BB
Size [nm]
C
Gal-3
4-1BB
4-1BB + Gal-3
Size [nm]

Figure EV3

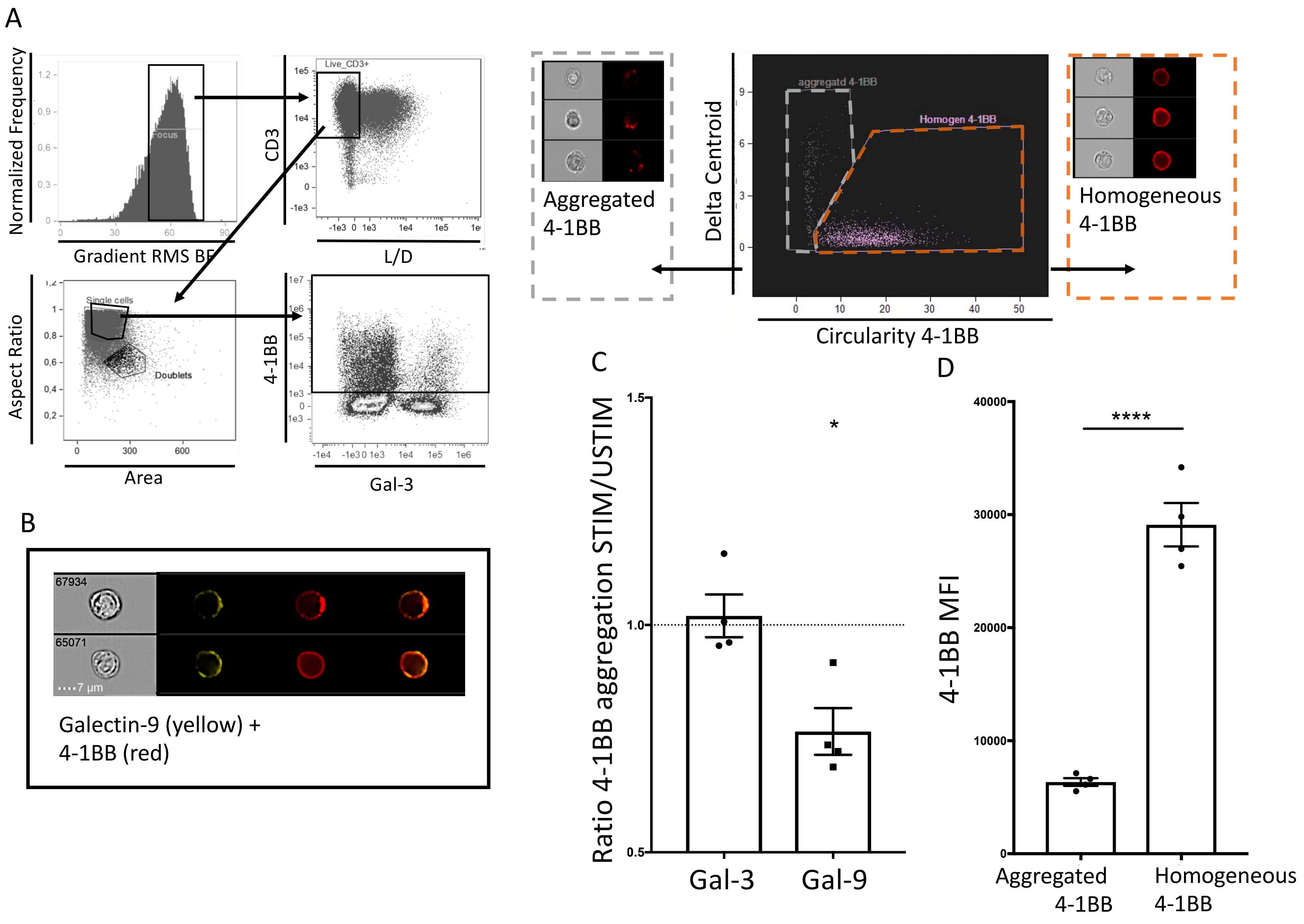
A
Normalized Frequency
Gradient RMS BF
Focus
CD3
L/D
Live_CD3+
Aspect Ratio
Area
Single cells
Doublets
4-1BB
Gal-3
Aggregated 4-1BB
Delta Centroid
aggregatd 4-1BB
Homogen 4-1BB
Circularity 4-1BB
Homogeneous 4-1BB
B
67934
65071
7 µm
Galectin-9 (yellow) +
4-1BB (red)
C
Ratio 4-1BB aggregation STIM/USTIM
*
Gal-3
Gal-9
D
4-1BB MFI
****
Aggregated 4-1BB
Homogeneous 4-1BB

Figure EV4

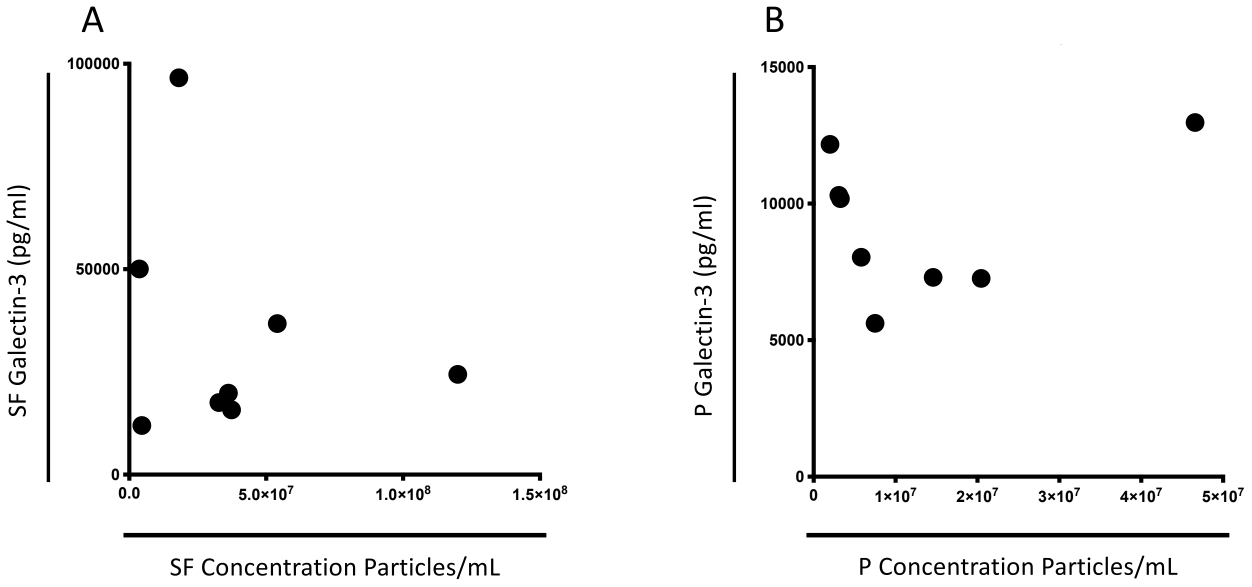
A
B
100000
50000
0
0.0
5.0×10^7
1.0×10^8
1.5×10^8
SF Galectin-3 (pg/ml)
SF Concentration Particles/mL
15000
10000
5000
0
0
1×10^7
2×10^7
3×10^7
4×10^7
5×10^7
P Galectin-3 (pg/ml)
P Concentration Particles/mL

Figure EV5

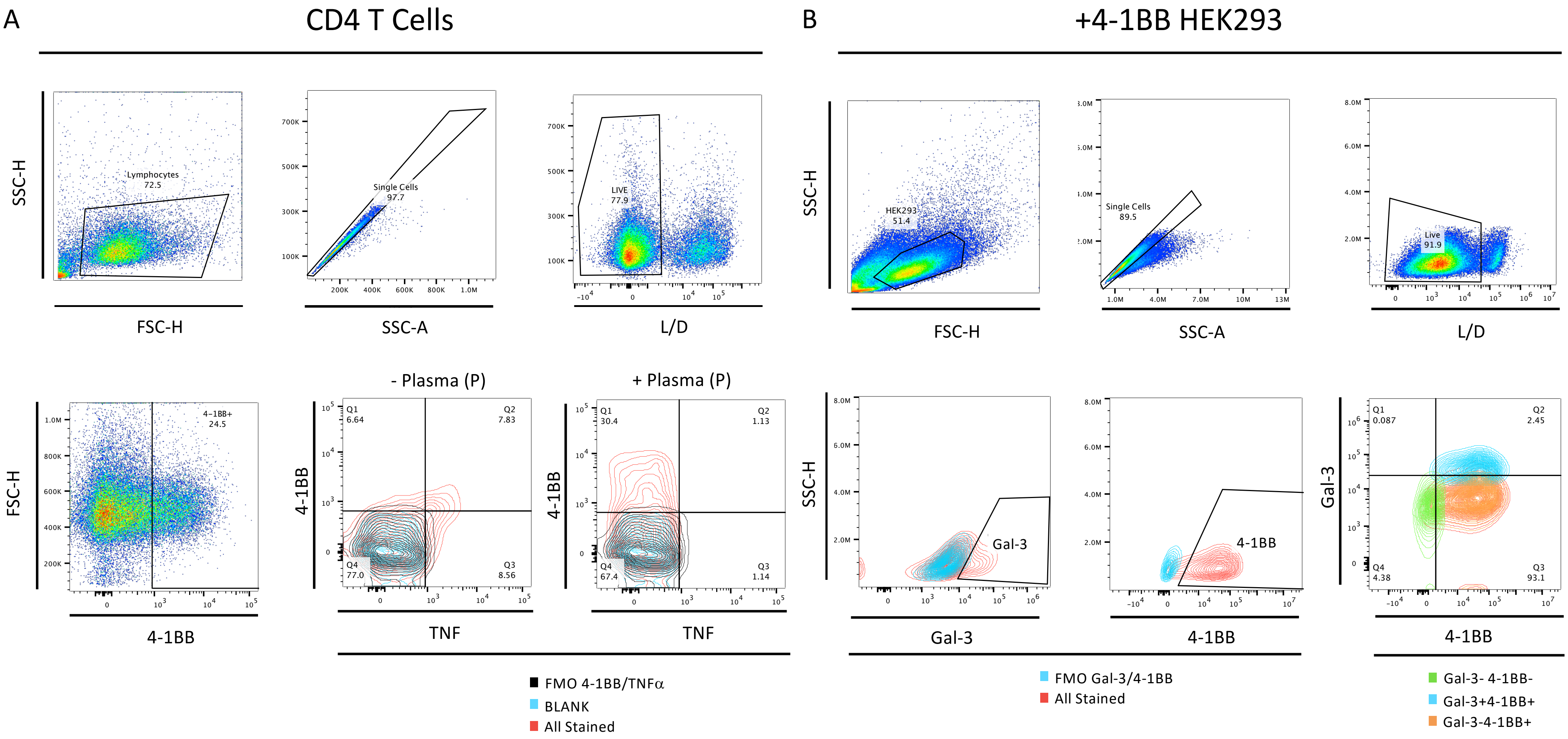

A
CD4 T Cells
B
+4-1BB HEK293
SSC-H
FSC-H
Lymphocytes
72.5
Single Cells
97.7
SSC-A
LIVE
77.9
L/D
FSC-H
4-1BB+
24.5
4-1BB
- Plasma (P)
+ Plasma (P)
4-1BB
TNF
Q1 6.64
Q2 7.83
Q3 8.56
Q4 77.0
Q1 30.4
Q2 1.13
Q3 1.14
Q4 67.4
FMO 4-1BB/TNFα
BLANK
All Stained
HEK293
51.4
Single Cells
89.5
Live
91.9
Gal-3
4-1BB
Gal-3
Q1 0.087
Q2 2.45
Q3 93.1
Q4 4.38
FMO Gal-3/4-1BB
All Stained
Gal-3- 4-1BB-
Gal-3+4-1BB+
Gal-3-4-1BB+

Figure EV6

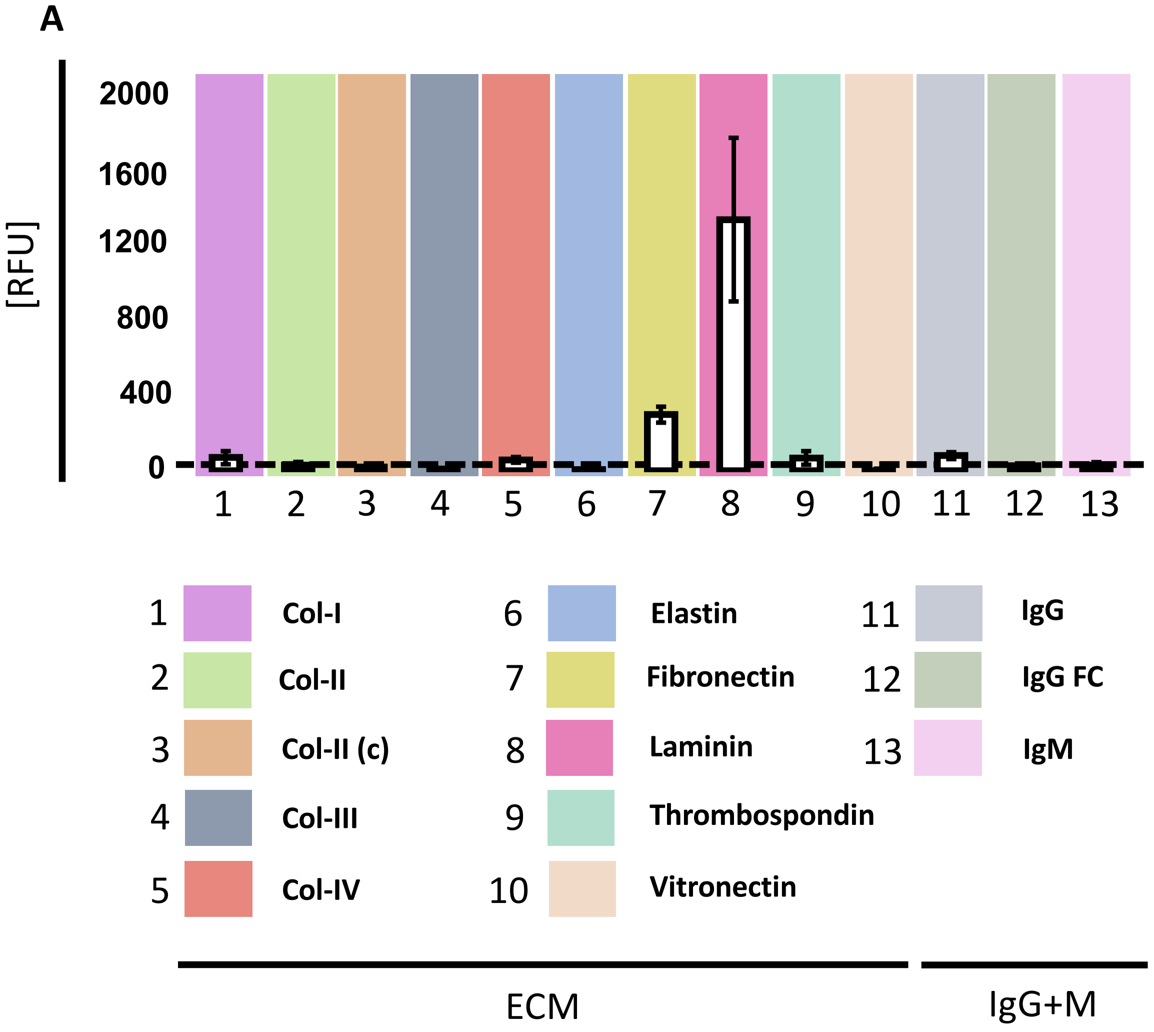
A
[RFU]
2000
1600
1200
800
400
0
1
2
3
4
5
6
7
8
9
10
11
12
13
1 Col-I
2 Col-II
3 Col-II (c)
4 Col-III
5 Col-IV
6 Elastin
7 Fibronectin
8 Laminin
9 Thrombospondin
10 Vitronectin
11 IgG
12 IgG FC
13 IgM
ECM
IgG+M

Figure EV7

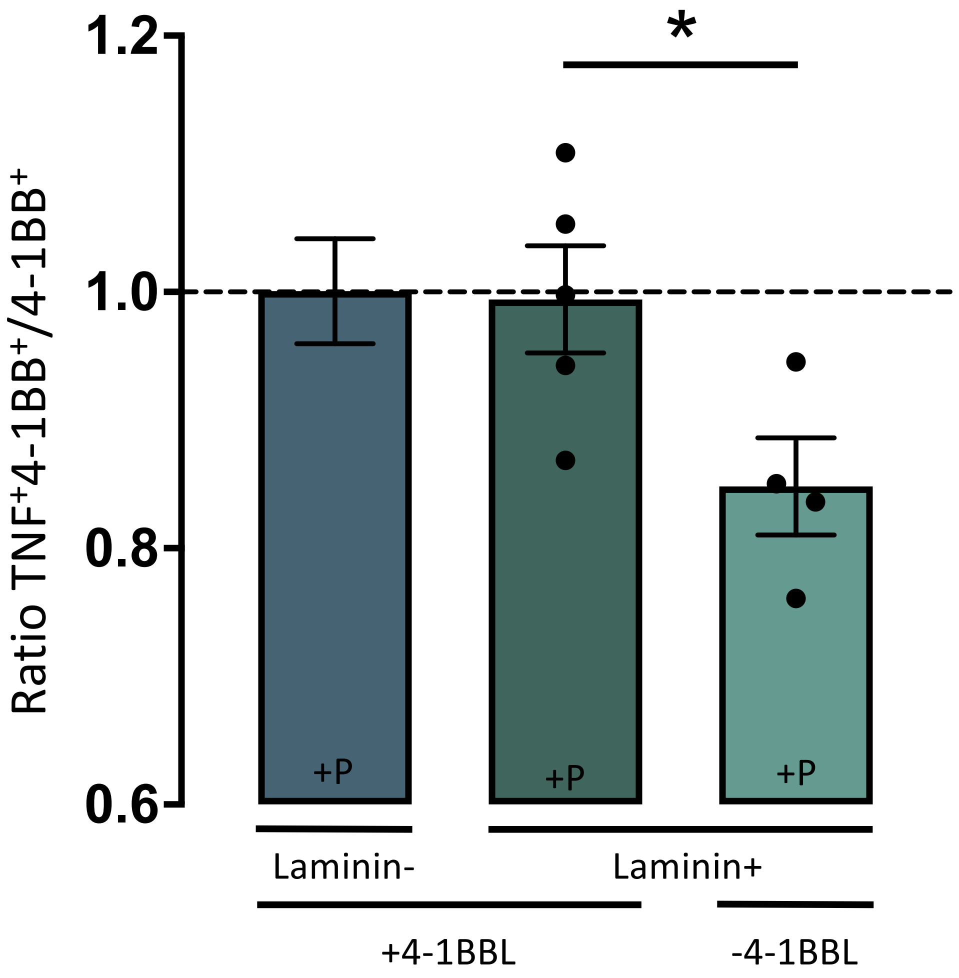
Ratio TNF⁺4-1BB⁺/4-1BB⁺
1.2
1.0
0.8
0.6
*
+P
+P
+P
Laminin-
Laminin+
+4-1BBL
-4-1BBL

Table EV1

Galectin	Rh4-1BB		RhFc	
	K_d (µM)	± SEM	K_d (µM)	SEM
-1 C3S	3,21	0,34	NT	N/A
-2	6,12	1,23	NT	N/A
-3	1,79	0,24	NB	N/A
-3 R186S	NB	N/A	NT	N/A
-8N	3,59	0,40	NT	N/A
-8C	NB	N/A	NT	N/A
-9N	1,39	0,26	NB	N/A
-9C	2,24	0,35	NB	N/A

Table EV2

Patient characteristics		
Diagnosis		RA = 20
Disease duration (years)		10 (1-33)
Sero-status (%)	S.pos/S.neg	63/37
DAS28CRP (0-10)		3.6 (3.2-4.9)
Gender (%)	Male/Female	25/75

Table EV3

ID	Designation	hGal-3 50 ug/ml		hGal-9 50 ug/ml	
		Ave	SD	Ave	SD
1	A2G0-ASN	1	2	0	70
2	224A3G0-ASN	0	2	0	30
3	226A3G0-ASN	0	2	55	337
4	A4G0-ASN	32	11	0	75
5	A2G0F-ASN	0	5	0	43
6	224A3G0F-ASN	1	1	0	84
7	226A3G0F-ASN	3	4	0	24
8	A4G0F-ASN	1	2	0	66
9	A2G2-ASN	273	272	10069	427
10	224A3G3-ASN	17695	4947	14481	2452
11	226A3G3-ASN	1011	461	11023	1425
12	A4G4-ASN	29047	6236	18753	1331
13	A2G2F-ASN	744	323	10213	2046
14	224A3G3F-ASN	17090	6449	15216	494
15	226A3G3F-ASN	2204	741	14866	1773
16	A4G4F-ASN	25246	959	23442	4982
17	2,3SA2-A2G2-ASN	131	32	256	76
18	2,3SA3-224A3G3-ASN	14881	5753	1387	233
19	2,3SA3-226A3G3-ASN	677	360	654	149
20	2,3SA4-A4G4-ASN	17921	5173	1955	140
21	2,3SA2-A2G2F-ASN	111	12	309	79
22	2,3SA3-224A3G3F-ASN	7005	4931	705	51
23	2,3SA3-226A3G3F-ASN	332	188	190	69
24	2,3SA4-A4G4F-ASN	17684	8039	869	115
25	2,6SA2-A2G2-ASN	1	5	0	51
26	2,6SA3-224A3G3-ASN	1	4	0	67
27	2,6SA3-226A3G3-ASN	3	2	26	23
28	2,6SA4-A4G4-ASN	3	2	0	62
29	2,6SA2-A2G2F-ASN	2	8	15	76
30	2,6SA3-224A3G3F-ASN	6	22	0	58
31	2,6SA3-226A3G3F-ASN	6	6	56	144
32	2,6SA4-A4G4F-ASN	0	3	0	28
33	Man3-ASN	0	2	0	37
34	Man5-AEAB	103	63	1068	144
35	NA2-AEAB	163	81	6270	387
36	2,6SA-NA2-AEAB	36	34	0	49
37	LSTc	216	136	18017	372
38	LSTd	203	38	907	48
